# Targeted fluorescence lifetime probes reveal responsive organelle viscosity and membrane fluidity

**DOI:** 10.1371/journal.pone.0211165

**Published:** 2019-02-14

**Authors:** Ida Emilie Steinmark, Arjuna L. James, Pei-Hua Chung, Penny E. Morton, Maddy Parsons, Cécile A. Dreiss, Christian D. Lorenz, Gokhan Yahioglu, Klaus Suhling

**Affiliations:** 1 Department of Physics, King’s College London, London, United Kingdom; 2 Randall Centre for Cell and Molecular Biophysics, King’s College London, London, United Kingdom; 3 Institute of Pharmaceutical Science, King’s College London, London, United Kingdom; 4 Department of Chemistry, Imperial College London, London, United Kingdom; University of Bath, UNITED KINGDOM

## Abstract

The only way to visually observe cellular viscosity, which can greatly influence biological reactions and has been linked to several human diseases, is through viscosity imaging. Imaging cellular viscosity has allowed the mapping of viscosity in cells, and the next frontier is targeted viscosity imaging of organelles and their microenvironments. Here we present a fluorescent molecular rotor/FLIM framework to image both organellar viscosity and membrane fluidity, using a combination of chemical targeting and organelle extraction. For demonstration, we image matrix viscosity and membrane fluidity of mitochondria, which have been linked to human diseases, including Alzheimer’s Disease and Leigh’s syndrome. We find that both are highly dynamic and responsive to small environmental and physiological changes, even under non-pathological conditions. This shows that neither viscosity nor fluidity can be assumed to be fixed and underlines the need for single-cell, and now even single-organelle, imaging.

## Introduction

Changes in cellular viscosity have been found in a range of human diseases, including cancer [1], Alzheimer’s disease [2], Huntington’s disease [3], Amyotrophic lateral sclerosis [3], and Leigh’s syndrome [4], as well as during disease treatment [5]. On a macroscopic level, viscosity is defined as the resistance to flow, but on the microscopic level—where the probe is comparable in size to the solvent molecules—it can be described, after some simplification, as the extent of free volume between solvent molecules and around the probe [6].

Only recently have the potentially extensive implications of altered viscosity and fluidity for biological reactions on the molecular level been recognised [7]. Some biological reactions are diffusion-limited, meaning that diffusion is rate-limiting, but practically all have a diffusion-mediated step [8]. If the viscosity of the medium through which a chemical species must diffuse is greatly altered, this will have a significant effect upon reaction rate [9]. Indeed it is possible that, should the viscosity increase markedly, some reactions that were not previously diffusion-limited will become so. It is even conceivable that some reactions, as a result of a high viscosity and reduced diffusion, will be overtaken by other, competing reactions and hence not even take place. This could have a cascading effect with consequences for the whole cell, or potentially the whole organism. Additionally, some organisms such as bacteria are known to adjust their membrane viscosity responsively [10]. Therefore, the ability to accurately monitor and visually follow changes in viscosity and fluidity is greatly beneficial when trying to understand the kinetics and implications of biological reactions.

Since mechanical viscometers cannot probe cellular viscosity, fluorescence-based methods have been developed, most notably fluorescence anisotropy [11]. This method requires polarisation-resolved data and specialist knowledge, and anisotropy imaging lacks commercial analysis software. The combination of fluorescent molecular rotors (FMRs) and fluorescence lifetime imaging (FLIM) is a recent alternative, providing easy and reliable imaging with viscosity as the contrast [7]. Using fluorescence lifetime instead of intensity is beneficial because intensity is concentration-dependent [12] but lifetime is not. This is particularly useful in biological samples where concentration homogeneity cannot be guaranteed. We believe that FMRs are a versatile solution and that they offer the potential for extending lines of inquiry within studies of disease and drug delivery.

**Fig 1:**
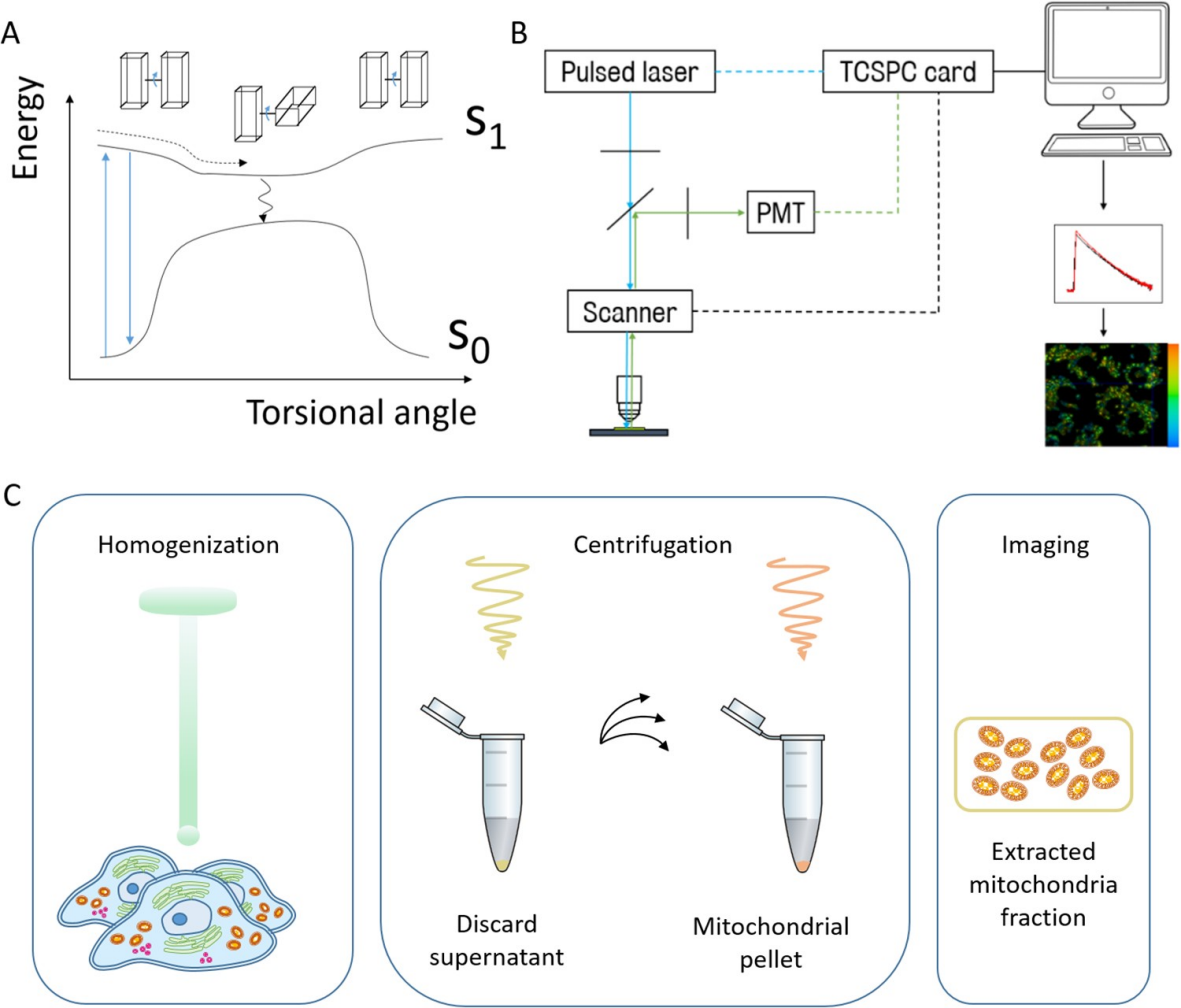
A) Schematic showing the concept behind FMRs, adapted from Levitt *et al* [13]. Normal absorption and fluorescence occurs when rotation is restricted, but a non-radiative pathway can be accessed when rotation is free. This influences the fluorescence lifetime. Note that while most FMRs are planar in the ground state, BODIPY rotors such as **FMR-1** and **FMR-2** are not [7]. B) Diagram of confocal FLIM microscope set-up using the scanning to get position and timing information. PMT: photomultiplier tube, TCSPC: time-correlated single photon counting. C) Diagram of the step involved in mitochondrial extraction.

FMRs are dyes that in the excited state can rotate around a single bond. This rotation gives access to a non-radiative deactivation pathway, such that in low viscosity environments the FMR will be very weakly fluorescent with a short lifetime (see Fig 1A). Conversely, in high viscosity environments, rotation will be restricted, thereby increasing the fluorescence and prolonging the lifetime [13],[7].

Cells contain many microenvironments that are often determinants of disease [14], so tools to investigate both organellar viscosity and membrane fluidity are sorely needed. This requires specific targeting of organelles, ideally with sub-organellar specificity for lumena, matrices and membranes.

One approach uses covalent bonding of an FMR to a protein [15]. This provides very versatile targeting and means only one rotor (with a suitable ligand) must be synthesised. However, the binding to such a comparably large structure lowers the viscosity sensitivity compared to a standard FMR, and such a framework cannot be used for membrane fluidity probing, as the FMR would not be able to embed into the membrane structure.

Instead we use a combination of chemical targeting and organelle extraction. Targeting is achieved with a small chemical motif that will not interfere with sensitivity and ensures diffusion through the targeted region. A range of targeting motifs already exist, such as triphenyl phosphonium (TPP+) for mitochondria [16], morpholine for lysosomes [17] and benzyl boronate [18] for the nucleus. Due to the high number of membranes in cells, specific probing of organelle membranes is challenging but can be achieved with extraction for most organelles (see Fig 1B) [19],[20].

Here, we focus on mitochondria. Mitochondrial matrix diffusion has been studied with steady-state anisotropy [21], fluorescence correlation spectroscopy (FCS) [4] and fluorescence recovery after photobleaching (FRAP) [22], and has been linked to complex I deficiency and Leigh’s syndrome. Furthermore, a few matrix-targeting lifetime FMRs have been reported [16],[23],[24]. Changes in mitochondrial membrane fluidity have been studied with steady-state anisotropy and linked to a range of neurodegenerative diseases and ageing [2],[3]. However, the mitochondrial membrane fluidity has never to our knowledge been imaged on a single organelle level, and potential dynamic and/or responsive viscosity and fluidity in non-diseased organelles is an active area of research [10]. Studying the viscosity response in healthy cells is crucial as we cannot make sound inferences about pathology without understanding the non-pathological baseline. Further, several vital antioxidant enzymatic systems in mitochondria are diffusion-limited [8],[25]. Therefore a change in viscosity could potentially have a big impact on their function [10]. This adds to the importance of studying the viscosity/diffusion aspects of mitochondria.

**Fig 2:**
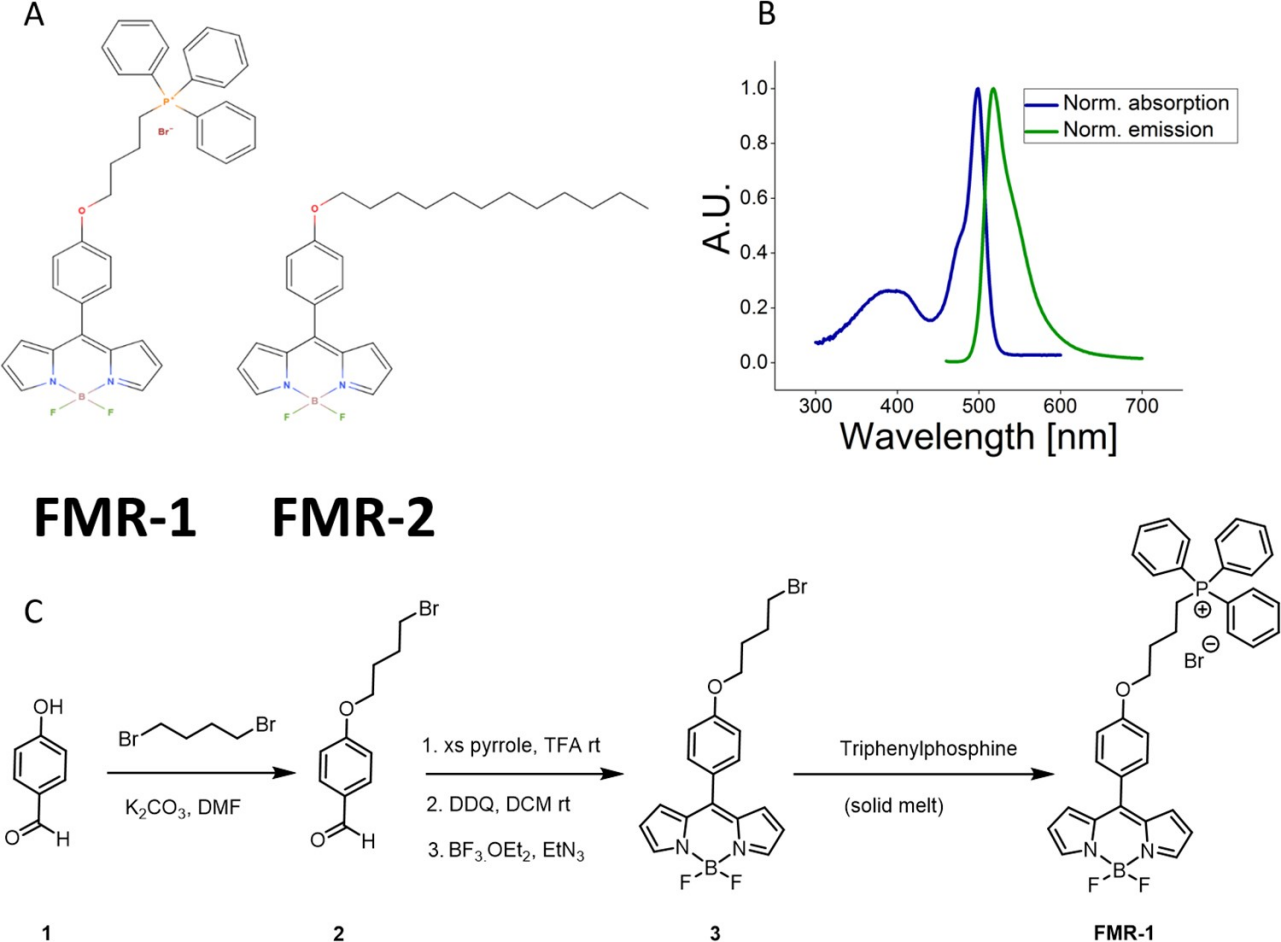
A) Chemical structure of the two FMRs used. **FMR-1** is the mitochondria-targeting BODIPY-based FMR and a TPP+ moiety, in the main text referred to as **FMR-1**. **FMR-2** is a BODIPY-based FMR with a C12 carbon chain [26], used to image the mitochondrial membrane fluidity, in the main text referred to as **FMR-2**. B) Normalised emission and absorption spectra of **FMR-1** in 80% glycerol and 20% methanol. Note that, while emission increases with viscosity, the shape of the spectrum remains unaltered. Absorption remains the same. C) Synthetic scheme for the synthesis of **FMR-1**. The synthesis of **FMR-2** can be found in previous publications [26].

We introduce a new, chemically-targeted FMR, **FMR-1** (see Fig 2), to image matrix viscosity and to investigate the latent effect of varying, non-pathological Ca^2+^ exposure. Next, we extract mitochondria from HeLa cells and image membrane fluidity using a well-known FMR, BODIPY-C12 or **FMR-2**, this time exploring the effect of different nutrient conditions during cell growth. The approach outlined in this paper provides a complete framework for imaging both organellar viscosity and fluidity, with applications in both clinical and biophysical studies.

## Materials and methods

### Chemical synthesis and characterisation

Synthesis and characterisation of **FMR-2** were described previously [26].

For the synthesis of **FMR-1**, please refer to Fig 2. All chemicals including anhydrous solvents were used as received from commercial suppliers. Analytical thin layer chromatography (TLC) was carried out on glass backed silica gel 60 GF254 (Merck©) plates and flash column chromatography was either performed on silica gel 60 (Merck©) or via automated chromatography (Biotage Isolera) using SNAP KP-Sil columns with the indicated solvent system.

Nuclear magnetic resonance (NMR) spectra were recorded on 500 MHz spectrometers at ambient probe temperature with predeuterated solvents as internal standards. ^19^F NMR spectra are reported in parts per million referenced to hexafluorobenzene. Mass spectra were carried out using ElectroSpray Ionisation (ESI) and only molecular ions are reported.

4-(4-bromobutyloxy)benzaldehyde **2** was synthesised according to a previous publication [27] but using anhydrous DMF as solvent.

#### Synthesis of FMR-1

Synthesis of BODIPY **3** follows a previous description [28]. The benzaldehyde **2** (1 g, 3.89 mmol) was dissolved in freshly distilled pyrrole (13.5 ml, 195 mmol) and the solution degassed by bubbling N_2_ for approximately 20 minutes before the addition of TFA. The mixture was stirred for 45 minutes at room temperature, diluted with DCM (100 ml) and washed consecutively with water (100 ml), NaHCO3 (100 ml, 0.5 M) and water (100 ml). The DCM layer was separated, dried over MgSO4, filtered and evaporated to remove the DCM, excess pyrrole was removed by high vacuum to give the dipyrromethane as a green viscous oil. TLC [Silica gel: DCM/Hexane 2:1] visualisation using Bromine vapour. The crude was purified by Biotage automated chromatography [DCM/Hexane 2:1] giving the pure dipyrromethane as a viscous green-yellow oil 0.75 g (51.7%). The dipyrromethane (0.75 g, 2 mmol) was dissolved in DCM (60 ml) and DDQ (0.46 g, 2 mmol) was added and the reaction stirred under N_2_, shielded from light for 45 minutes. Then triethylamine (0.87 ml, 5.88 mmol) was added followed immediately by the addition of BF_3_. (OEt_2_)_2_ (0.62 ml, 5.02 mmol) and the reaction was stirred at room temperature overnight. The reaction mixture was washed consecutively with water (100 ml), NH_4_Cl (100 ml, 0.5 M), NaHCO_3_ (100 ml, 0.5M) and finally with water (100 ml). The organic layer was dried over MgSO_4_, filtered and evaporated to give a dark viscous oil which was purified by Biotage automated chromatography [DCM/EtOAc] to give BODIPY **3** as an orange-green solid 0.13 g (15.5%) BODIPY **3** (0.1 g, 0.24 mmol) and triphenyphosphine TPP (0.19 g, 0.72 mmol) were reacted as a solid melt at 90°C, after 8 hrs TLC [Silica gel: DCM/Hexane 8:2] still showed the presence of BODIPY **3** as well as an orange fluorescent base-line spot which moved as a single spot on TLC [Silica gel: DCM/Acetone/MeOH 10:2:1]. A further portion of TPP (0.19 g, 0.72 mmol) was added and heating continued for a further 8 hrs after which the reaction was complete by TLC. The reaction was cooled to room temperature, resulting in a red-brown solid that was triturated with diethyl ether, filtered and dried to give a red-brown powder. This was purified by flash chromatography [Silica gel: DCM/Acetone/MeOH 10:2:1], the crude was loaded on top of the column as a concentrated solution in DCM, eluted with 5 column volumes of DCM resulting in the separate elution of both TPP and unreacted BODIPY **3**. FMR-1 was then eluted with DCM/Acetone/MeOH, coming off the column as a bright orange streaky band. Fractions were pooled and evaporated to give pure **FMR-1** as a bright orange solid 0.13 g (80%). ^1^H NMR (500 MHz, CDCl_3_) *δ*H 7.89-7.84 (m, 8H), 7.8-7.76 (m, 3H), 7.7-7.66 (m, 6H), 7.5 (d, J = 8.8 Hz, 2H), 7.01 (d, J = 8.8 Hz, 2H), 6.95 (d, J = 4.3 Hz, 2H), 6.53 (dd, J = 4.3, 1.9 Hz, 2H), 4.25 (t, J = 5.8 Hz, 2H), 4.06-3.95 (m, 2H), 2.28 (t, J = 6.4Hz, 2H), 1.9 (q, J = 7.8Hz, 2H); ^13^C NMR (500 MHz, CDCl_3_) *δ*C 161.34, 147.39, 143.29, 134.99, 134.75, 133.74, 132.45, 131.34, 130.49, 126.24, 118.66, 118.24, 117.98, 114.64; ^19^F NMR (500 MHz, CDCl_3_) *δ*F -145.11; MS (ESI) m/z found 601.2384 [M-Br]+ calculated for C_37_H_3_BF_2_N_2_OP, 601.2392.

### Calibration

Methanol/glycerol fractions ranging from 0 to 90% glycerol were used for the calibration, containing 13 *μ*M of **FMR-1**. Absorption spectra were collected on a Hitachi U4100 Emission Spectrometer, and emission and excitation spectra were collected using a Horiba FluoroMax-4 Spectrofluorometer. For all spectral measurements, Thorlabs 3500 *μ*L quartz cuvettes with four clear sides containing around 3mL of sample solution was used.

For quantum yield measurements, both PM546 (∼ 0.4 *μ*M, quantum yield 0.95) and Alexa Fluor488 (∼ 0.4 *μ*M, quantum yield 0.92) were used as reference dyes. Methanol was used as baseline for PM546 and FMR-1 (refractive index 1.3292), water for Alexa Fluor488 (refractive index 1.333). The quantum yield was calculated using

$$\begin{array}{c} QY=QY_{ref}\frac{n^{2}}{n_{ref}^{2}}\frac{I}{Abs}\frac{Abs_{ref}}{I_{ref}} \end{array}$$

Where n is the refractive index of the solvent, I is the integrated fluorescence intensity, and Abs is the absorbance at the excitation wavelength (kept between 0.02-0.05 to ensure linear response) [29].

**Fig 3:**
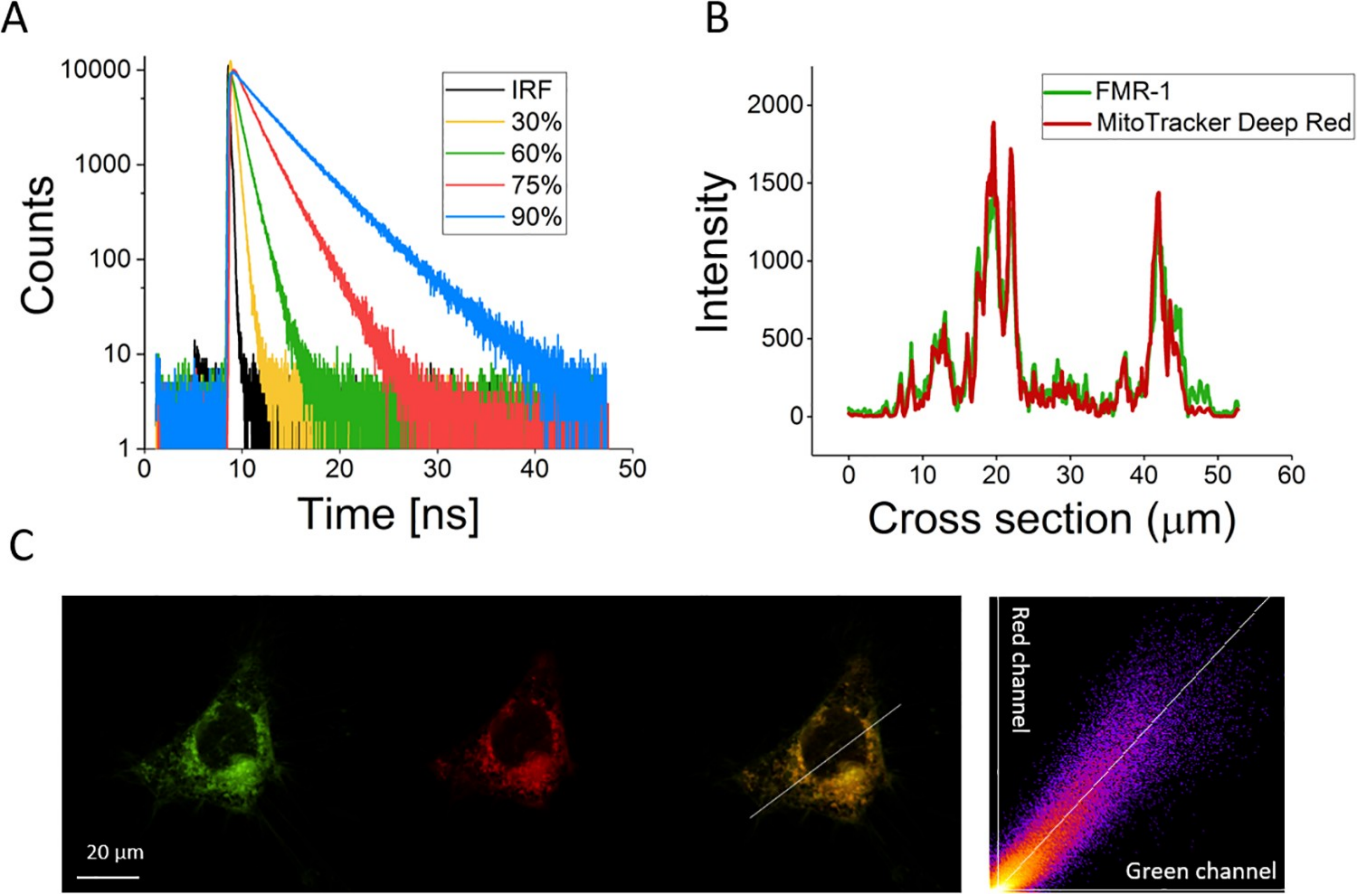
A) Representative single decays from **FMR-1** in methanol/glycerol fractions for calibration. B) Intensity correlation along the cross section of the merged image on the far right of Fig 3C. C) Confocal image of HeLa cell stained with both **FMR-1** and MitoTracker Deep Red, excited at 488 nm and 642 nm, respectively. On right, merged image with position of cross-section in Fig 3C indicated. Intensity correlation between red and green channel across the whole of the merged image on far right. Manders colocalisation coefficients *M*_1_ (green in red channel) and *M*_2_ (red in green channel) are 0.92 and 0.96, respectively.

Fluorescence lifetime measurements were conducted on an inverted TCS-SP2 microscope (Leica Microsystems, Germany) and SPC-150 TCSPC boards (Becker & Hickl, Germany). 200 *μ*L of sample solution was pipetted into a well in an uncoated 8-well plate from Ibidi (Integrated BioDiagnostics). The instrument response function (IRF) was made from NaI-quenched fluorescein/methanol solution. A pulsed 467 nm diode laser (Hamamatsu) with a 20 MHz repetition rate and 90 ps optical pulse width was used to excite the samples (see Fig 3).

Lifetime measurements controlling for effects from polarity (dielectic constant), ionic strength and temperature were carried out in a similar fashion. For polarity measurements, solutions of 70% glycerol and either 30% methanol, ethanol, butanol or isopropyl alcohol were used. For ionic strength measurements, increasing concentrations of NaCl (from 0 to 0.26 mol) in 70% glycerol, 30% methanol were used. For temperature controls, 100% glycerol was heated and measured at 22°C, 30°C and 37°C using a microscope stage incubator.

Bulk viscosity measurements for the control experiments were carried out on a strain-controlled rheometer ARES (TA Instruments), equipped with a 25 mm parallel plate geometry and a temperature-controlling Peltier unit (C-PTD 200), with a gap between 0.6 and 0.9 mm. As an exception, the viscosity values for the 100% glycerol sample at different temperatures were calculated using an open-science GUI based on parameterisation from literature [30][31].

### Cell work

HeLa cells were cultured in a 12.5 mL petri dish (Thermo Fisher Scientific) in DMEM with 10% FBS, 1% 1X non-essential amino acid, 1 mM sodium-pyruvate and 0.1% penicillin/streptomycin at 37°C in a Hela Cell 150 incubator from Thermo Electron Corporation with 5% CO_2_. A few days before imaging, a sterile and coated 8-well plate (Ibidi) was prepared. Cells were harvested using 3 mL 1X Trypsin/EDTA and 7 ml complete DMEM. On the day of imaging, cells were washed three times with fluorescence imaging medium (FluoroBrite DMEM, Thermo Fisher Scientific), before staining with **FMR-1** (well concentration 1 *μ*M). Cells were incubated for 30 min. A similar protocol was followed for HCE cells, generously gifted by Struan Bourke from King’s College London and Min S Chang from Vanderbilt University [32].

### Extraction of mitochondria

Mitochondrial extraction was done according to established protocol [19]. This mainly includes stages of homogenization and centrifuging at ice-cold temperatures using an iso-osmotic homogenization buffer and a hypotonic buffer. The protocol uses cell pellets. In this case, cells were pelleted by washing in PBS-CMF twice, harvesting them using 2 mL 1X Trypsin/EDTA and 8 mL of DMEM/F12 before transfer to a conical tube. Cells were then spun at 1400 rpm at 4°C for 5 minutes before the supernatant was decanted. A second round of centrifuging and decanting followed re-suspension in PBS-CMF. Extracted mitochondria were imaged by staining them with **FMR-2** (including 30 min incubation) before suspending them in an agarose-gel scaffold. This was achieved by adding 0.3% agarose to a hypotonic buffer, bringing the solution to the boil to dissolve the agarose and while still hot to the touch, adding the extracted mitochondria such that the gel set around them. This allowed them to be kept in place during imaging. It should be noted that the mitochondrial fraction may contain some other membrane domains, but only structures of the right shape and size were analysed.

### Mitochondrial perturbation

Two methods were used to perturb the mitochondrial matrix viscosity. Firstly, HeLa cells were treated with an ionophore with no uncoupling activity, nystatin. Nystatin was dissolved in methanol at 28°C before being diluted into FluoroBrite DMEM (ThermoFisher Scientific) for cell treatment. Cells were incubated with a 10 *μ*M well concentration for 30 min prior to staining with **FMR-1**. Secondly, HeLa cells were treated with histamine which induces Ca^2+^ release from the endoplasmic reticulum to the mitochondria. Histamine was dissolved in de-ionised water and diluted in FluoroBrite DMEM for cell treatment. Prior to staining, cells were incubated for 30 min.

### Co-localisation

HeLa cells were pre-stained with MitoTracker Deep Red (Thermo Fischer Scientific) with a well concentration of 0.5 *μ*M. The cells were incubated for 30 min. The colocalisation imaging was done on a Nikon inverted confocal scanning microscope using a 488 nm and a 642 nm diode laser (carried out at the Nikon Imaging Centre at King’s College London), see Fig 3C.

### FLIM experiments

Cells were imaged in 8-well plates on a inverted TCS-SP2 microscope (Leica Microsystems, Germany) and SPC-150 TCSPC boards (Becker & Hickl, Germany). **FMR-1** and **FMR-2** were excited using a picosecond-pulsed (90 ps optical pulse width, 20 MHz repetition rate) 467 nm diode laser (Hamamatsu, Japan) through a 63X 1.2 NA water objective, with a 485 nm dichroic mirror and a 500 long pass filter. All imaging was done at room temperature. Each FLIM image was acquired over 900 s to ensure as little noise as possible (much shorter acquisition rates are possible). Lifetime histograms and FLIM images were analysed using SPCImage software (Becker & Hickl, Germany).

### Statistics

Welch-corrected two-tailed two-sample t-tests were used to estimate statistical significance. For the control *versus* nystatin experiment, the t-value was -9.3 and had 46.8 degrees of freedom. For the low *versus* high glucose extracted mitochondria experiment, the t-value was -3.2 and had 40.6 degrees of freedom.

### Molecular dynamics simulations

As we have done previously for the environmentally sensitive fluorescent dye, PRODAN [33], we have used all-atom classical molecular dynamics (MD) simulations to investigate the effect that the **FMR-2** dye has on the molecular scale properties of a model lipid bilayer. Therefore, two systems were simulated: (a) a DOPC bilayer consisting of 288 lipid molecules (144 molecules in each leaflet) and (b) the same bilayer interacting with a single **FMR-2** molecule. The lipid bilayer was built using the CHARMM-GUI membrane builder [34–36]. The membrane was first minimised using a steepest descent algorithm in order to remove any steric clashes that might exist in the structure obtained from the membrane builder. Then a series of equilibration simulations were carried out in the NVT (constant Number, Volume and Temperature) ensemble to equilibrate the temperature at 303.15 K and in the NPT (constant Number, Pressure and Temperature) ensemble to equilibrate the volume of our simulation system at a temperature of 303.15 K and a pressure of 1 atm. Finally, the DOPC bilayer system was simulated for a further 100 ns in the NPT ensemble at a temperature of 303.15 K and a pressure of 1 atm.

Then we inserted a single **FMR-2** molecule into the aqueous phase of the final configuration of this 100 ns simulation of the DOPC bilayer, such that it was at least 1.0 nm away from the interface of the bilayer. Then this system was run for 200 ns using the NPT ensemble at a temperature of 303.15 K and a pressure of 1 atm. The **FMR-2** molecule inserted into the bilayer within the first 100 ns of the simulation. Then the final 100 ns of this simulation were used for the analysis that was carried out. Likewise, the DOPC bilayer system (without the **FMR-2** molecule) was simulated for a further 100 ns, which was used for all analysis of it.

All of the simulations were carried out using the GROMACS molecular dynamics package [37, 38]. The interactions of the lipid molecules are described by the CHARMM36 lipid forcefield [39]. The interactions of the **FMR-2** molecule were modeled using the parameters for the BODIPY head group that had been previously used [40], and then the rest of the molecule was parameterised using the CHARMM General Force Field [41]. The water molecules and their interactions were modeled using the CHARMM version of the TIP3P model [42]. In all cases, the nonbonded interactions were cut-off at 1.0 nm, and the PME algorithm was used to account for long range electrostatic interactions. The LINCS algorithm was used to constrain all hydrogen-containing bonds, and a 2 femtosecond timestep was used during the production simulations. In all simulations, we used the Nosé-Hoover thermostat to control the temperature and the Parrinello-Rahman barostat to control the pressure.

In order to investigate the dynamics of the lipid molecules within each bilayer, we calculated the mean square displacement (MSD) in the *x*-*y* plane of the phosphorous atoms in the phosphatidylcholine headgroup of the DOPC lipid molecules. The mean square displacement is calculated as follows:

$$\begin{array}{c} MSD=\frac{1}{N}\Sigma_{n=1}^{N}\left({\left(x_{n}\left(t\right)-x_{n}\left(t_{ref}\right)\right)}^{2}+{\left(y_{n}\left(t\right)-y_{n}\left(t_{ref}\right)\right)}^{2}\right) \end{array}$$ (1)

where *N* is the number of atoms in your system, *x*_*n*_(*t*) and *y*_*n*_(*t*) are the *x* and *y* coordinates of atom *n* at time *t*, and *x*_*n*_(*t*_*ref*_) and *y*_*n*_(*t*_*ref*_) are the *x* and *y* coordinates of atom *n* at a reference time *t*_*ref*_.

The structure of the lipid membrane is characterised by the calculating the carbon-hydrogen lipid order parameter for both the *s*n-1 and the *s*n-2 chains of each lipid molecule. The lipid order parameter is calculated by:

$$\begin{array}{c} S_{CD}=\frac{1}{2}\left(3\cos^{2}\beta -1\right) \end{array}$$ (2)

where *β* is the angle that each C-H bond makes with the vector normal to the bilayer. The larger in magnitude that *S*_*CD*_ is, the more ordered the lipid molecules are. We have calculated this quantity for each molecule. Then each lipid was placed into one of five different distance (in *x*-*y* plane) ranges (*d* < 0.7 nm, 0.7 nm < *d* < 1.0 nm, 1.0 nm < *d* < 1.6 nm, 1.6 nm < *d* < 2.0 nm, *d* > 2.0 nm), where the smallest range represents the distance of first neighbours as determined from the radial distribution function of phosphorous atoms in the PC headgroup around the boron atom in the **FMR-2** molecule (not shown). We then average this quantity for each carbon in each tail over time and over each molecule within a given distance range.

Finally, we measured the hydration of the phosphatidylcholine group as an indication of the interfacial properties of the lipid membrane. In order to do so, we determined the radial distribution functions of the oxygen atoms in water molecules around the nitrogen atom in the onium group of the PC headgroup, the phosphorous atom in the phosphate group of the PC headgroup and the double bonded oxygens in the ester groups of the PC headgroup (not shown). From these radial distribution functions, we identified the distance at which first neighbour water molecules are found. This distance was used to then count the number of water molecules around each of these three groups of each lipid headgroup. The lipids were once again placed into one of the previously stated five different distance ranges. Then we averaged the number of water molecules found around each group of the lipid headgroup as a function of time and the number of lipids within each distance range.

## Results

### Validation and co-localisation of FMR-1

**FMR-1** is based on a simple BODIPY design, incorporating a mitochondria-targeting moiety, triphenylphosphonium (TPP+). For **FMR-1**, the fluorescence lifetime increases from just over 200 ps in 100% methanol (viscosity 0.6 cP at 22°C, quantum yield 2%) to 4.6 ns in 100% glycerol (viscosity 1178 cP at 22°C, quantum yield 55%). Low and high viscosity regions were fitted with straight lines according to the Förster-Hoffman equation, yielding gradients of 0.25 and 0.63 respectively. This two-slope behaviour is common for FMRs [7]. For a more in-depth explanation of the relationship between molecular rotors and viscosity, see the following references [7][6]. For our purposes, the theoretical relationship is less important; this is simply an empirical, observational relationship between fluorescence lifetime and viscosity. We additionally tested for temperature, polarity and ionic strength dependence in the relevant region of the calibration plot and found that, as expected, that **FMR-1** is only sensitive to viscosity. This is in line with previous publications on BODIPY FMRs [43][44].

It was co-localised with MitoTracker Deep Red in HeLa cells, yielding an average Pearson’s r of 0.92, as well as average Manders Colocalisation Coefficients (MCC) M_1_ 0.92 (green in red channel) and M_2_ 0.96 (red in green channel)—see Fig 3C. The average was taken over 16 co-stained cells. As is evident from the MCC, **FMR-1** stains slightly more regions than MitoTracker Deep Red, and judging by the images, this weak additional staining is in the plasma membrane which is not uncommon for this type of FMR [23].

**Fig 4:**
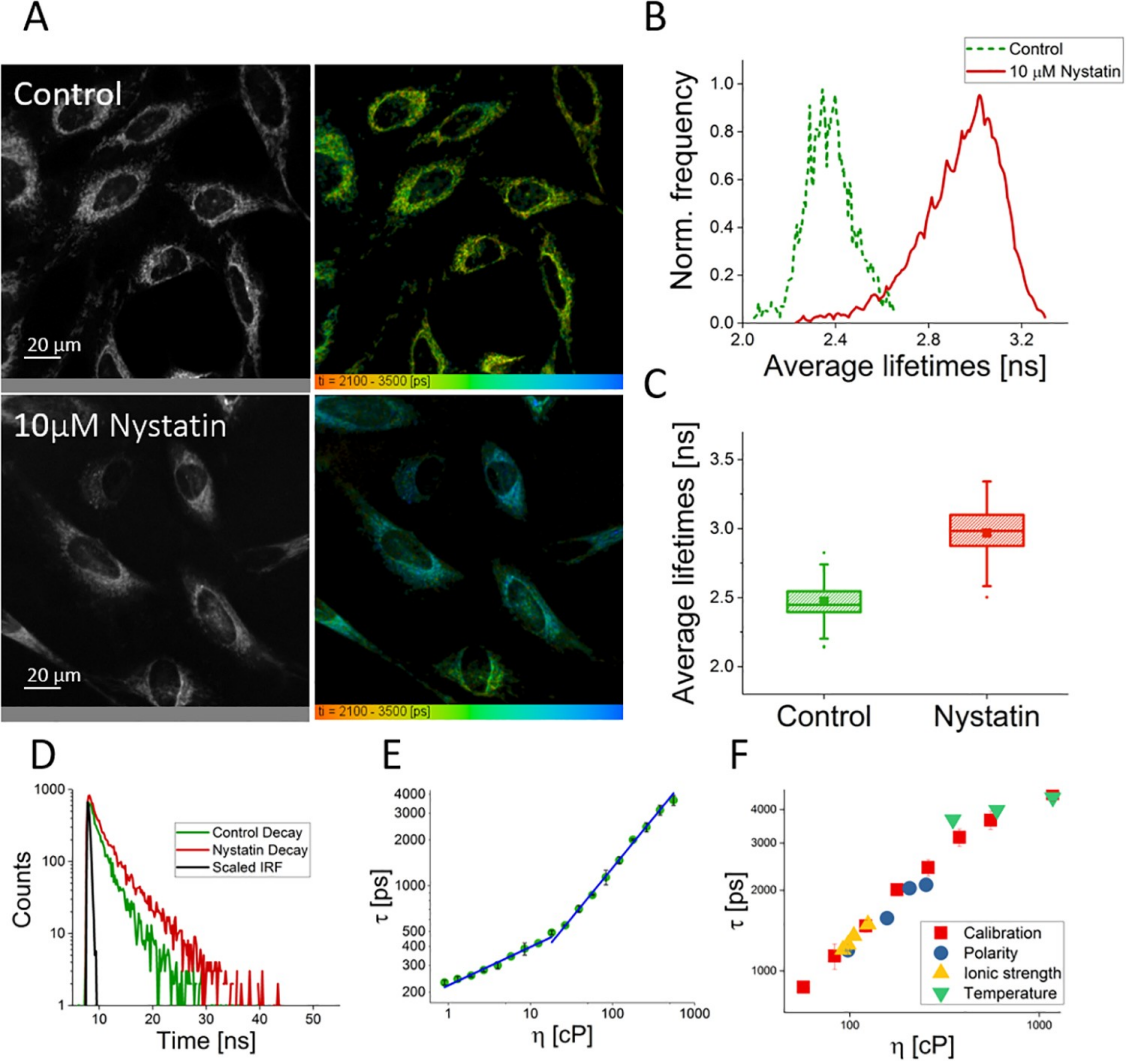
A) Representative fluorescence intensity and FLIM images of control and nystatin-treated HeLa cells, lifetime range 2100-3500 ps. B) Representative average fluorescence lifetime histograms from images A-B, control in green and nystatin-treated in red. C) Box plot showing the average lifetimes of cells in the control condition (n = 140, 1*μM* **FMR-1**) and nystatin-treated condition (n = 33, 1*μM* **FMR-1**). The average control viscosity was found to be 274.7 ± 54.4 cP, and the average nystatin-treated viscosity was found to be 367 ± 78.1 cP. The difference is statistically significant (*p* < 0.01.). D) Representative fluorescence intensity decays from a control cell (in green) and a cell treated with nystatin (in red), and a scaled instrument response function (IRF, in black). E) Calibration plot for **FMR-1** based on lifetimes in methanol-glycerol fractions. F) Control data for polarity, ionic strength and temperature plotted on top of the calibration data, in the relevant region of the plot.

FMR-FLIM of the HeLa cells showed that the viscosity of the mitochondrial matrix was around 275 cP at room temperature as calculated from the average lifetime (see Fig 4A). It is higher than that found using another BODIPY-based FMR (ca. 67.5 cP) [16]. This can be explained by a difference in temperature and the fact that Yang *et al*. [16] fit their entire calibration plot with one straight line, with a deviation from the data in the lower range—in which their viscosity value falls.

To validate the FMR, we compared the results to cells which had been pre-treated with nystatin (see Fig 4A). Nystatin is an ionophore with no uncoupling activity [45]. Ionophores are known to push mitochondria from the orthodox to the condensed ultrastructural state, which is dominated by high protein content and small volume, as evidenced by electron microscopy, and high matrix viscosity, as found by fluorescence anisotropy [21]. As such, we hypothesised that we would see an increase in matrix viscosity. After nystatin treatment, the matrix viscosity increased from 274.7 ± 54.4 cP to 367 ± 78.1 cP, as calculated from average lifetimes, which were 2473 ± 119 ps and 2967±185 ps respectively. This confirms **FMR-1**’s ability to measure changes in matrix viscosity.

### Matrix viscosity after Ca^2+^ exposure

Next we tested the effect of Ca^2+^ exposure on matrix viscosity. Ca^2+^ uptake into mitochondria is a normal, physiological process, but is still not fully understood [46]. As such, we wanted to explore any viscosity effects of non-pathological Ca^2+^ exposure. Histamine induces a short Ca^2+^ release from the endoplasmic reticulum to the mitochondria in HeLa cells at low concentrations (< 100*μM*), which lasts only a few minutes. We aimed to investigate subsequent viscosity effects as a result of the Ca^2+^, hence avoiding the Ca^2+^ influx itself.

**Fig 5:**
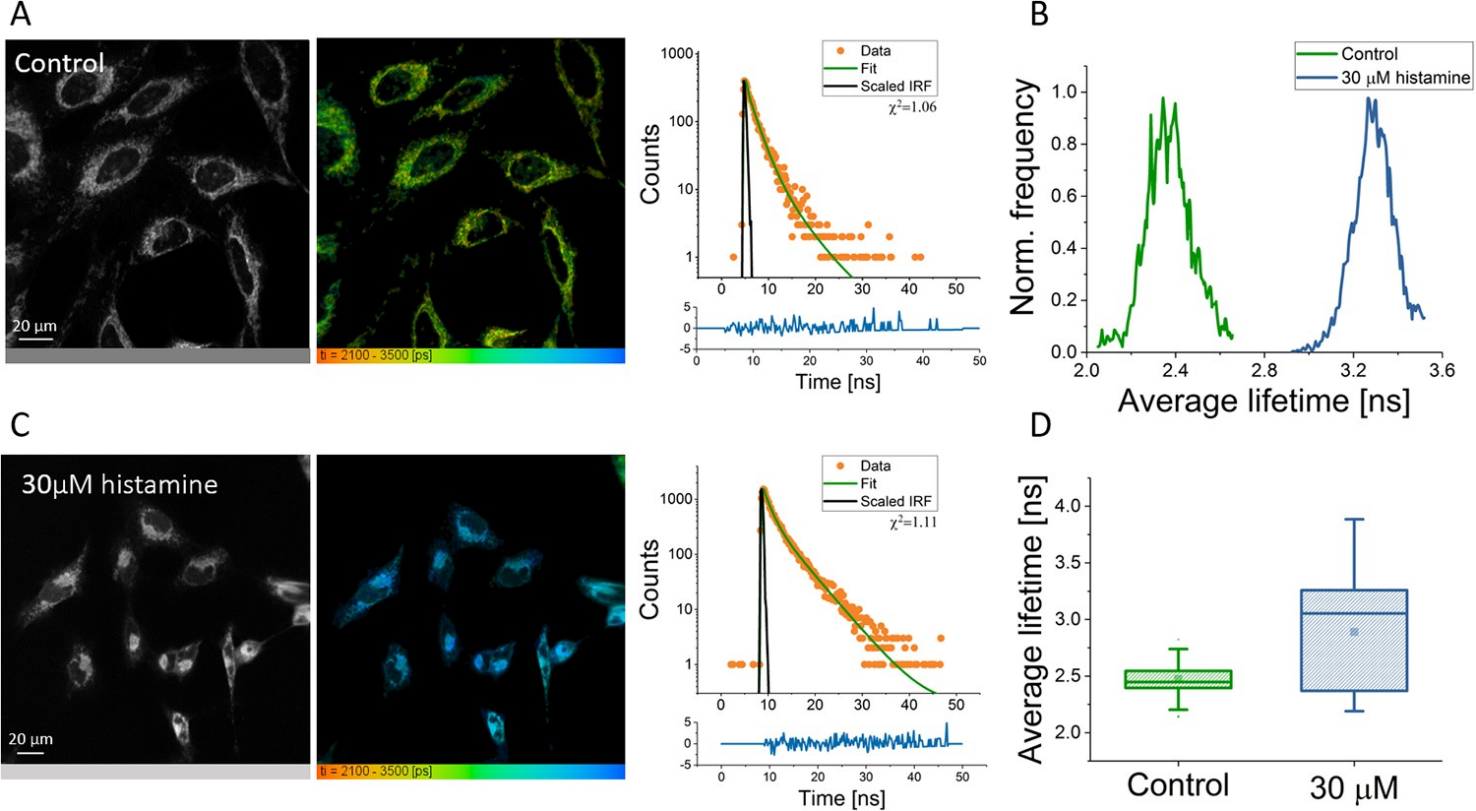
Representative fluorescence intensity and FLIM image of control HeLa cells (A) and of histamine-treated HeLa cells (C), along with representative decays, showing fit and scaled IRF. Lifetime range for images is 2100-3500 ps. 1*μ*M **FMR-1** in both conditions. B) Representative average fluorescence lifetime histograms from cells in each condition. D) Box plot showing the average lifetimes of cells in the control (n = 140) and the histamine condition (n = 83). Each data point is a cell.

We pre-treated HeLa cells with histamine prior to FMR staining in order to observe genuine viscosity changes, and not simply the influx of Ca^2+^. FMR-FLIM showed that after treatment with 30*μ*M histamine, the viscosity varied greatly and showed differences between individual cells (see Fig 5). At lower concentrations, histamine appears to have little to no effect on viscosity, but the variation is sustained at higher concentrations (see S2 Fig). This could possibly be due to cells responding differently depending on pre-existing conditions, such as stage in cell cycle. All in all, it shows the dynamic nature of the matrix viscosity, and that it is both responsive to small physiological changes and highly variable between cells. We conclude that matrix viscosity cannot be assumed to be fixed and underline the need for single-cell viscosity imaging.

### Effect of glucose on membrane fluidity

Previous studies of mitochondrial membrane fluidity, which have utilised steady-state anisotropy, have found that the membrane is ridigified in neurodegenerative diseases. This has been attributed to oxidative damage, leading to lipid peroxidation which in turn results in increased membrane rigidification [2],[3].

To explore membrane fluidity during a non-pathological process, we assessed the effect of nutrition, specifically glucose. Excess glucose leads to increased energy production which can result in higher reactive oxygen species (ROS) production [47], but conversely, starvation can also lead to increased ROS production [48],[49]. The effect of small perturbations in glucose on ROS, and hence fluidity, is therefore not obvious. Here, we explored the effect of high or low glucose conditions.

**Fig 6:**
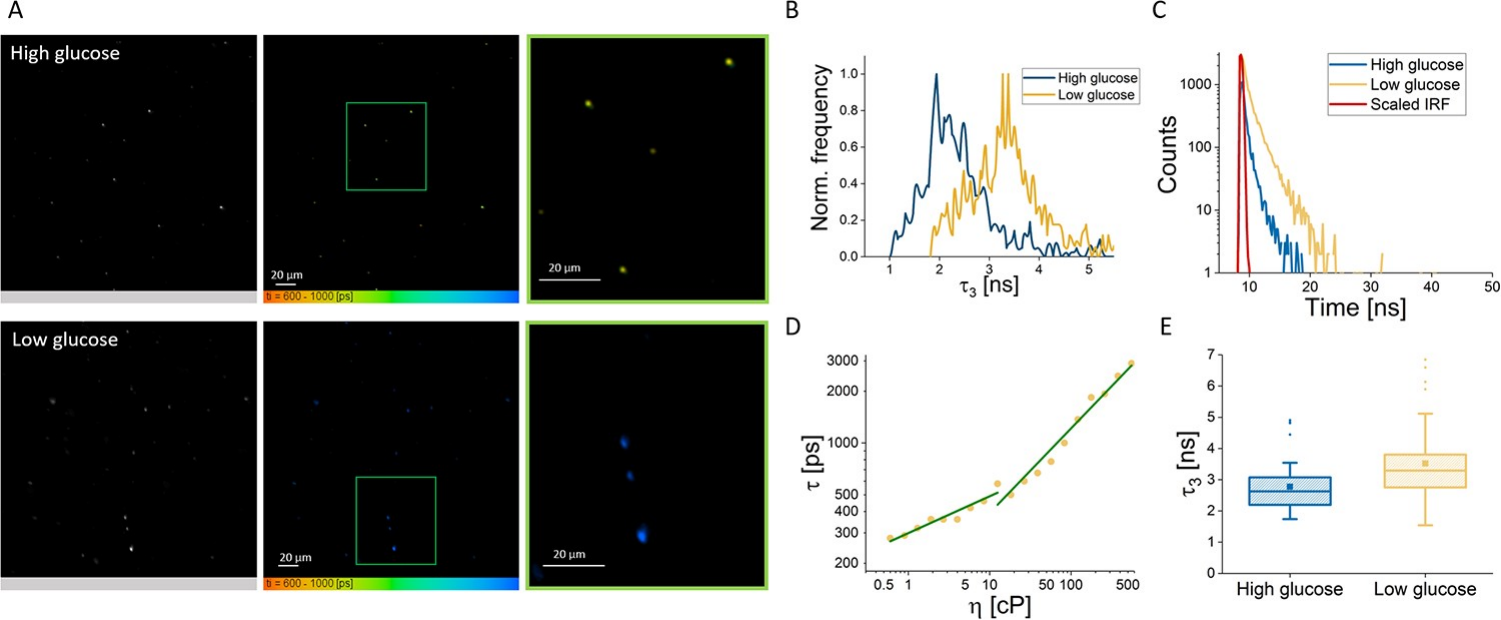
A) Representative fluorescence intensity and FLIM images of extracted mitochondria, originally grown as HeLa cells in high (4.5*g*/*L*) and low (1*g*/*L*) glucose DMEM respectively. Average lifetime range is 600-1000 ps. B) Histograms of the third lifetime component for the images in Fig 5A, high glucose condition in blue and low glucose condition in yellow. C) Representative fluorescence intensity decays from extracted mitochondria, high glucose in blue, low glucose in yellow and scaled IRF in red. D) Calibration plot for **FMR-2**, re-produced from P.H. Chung’s thesis [50]. E) Box plot showing the third lifetime components of mitochondria in the two conditions (n = 42 for high glucose and n = 48 for low glucose). The difference is statistically significant (*p* < 0.01). On average, the viscosity for the high glucose condition was found to be 536.8 ± 303 cP (from an average third component lifetime of 2775 ± 468 ps). For the low glucose condition, it was found to be 869.5 ± 659.8 cP (from average third component lifetime of 3517 ± 877 ps) on average.

HeLa cells were grown in either standard, high glucose DMEM (4.5*g*/*L*) or low glucose DMEM (1*g*/*L*) prior to extraction. Once extracted, they were then stained with the membrane-targeting **FMR-2**, suspended in a 0.3% agarose gel scaffold and imaged (see Fig 6). We found that the low glucose condition lead to an increase in lifetime, and hence a decrease in membrane fluidity, in accordance with trends observed for more extensive starvation [48],[49].

The change between the two conditions primarily took place in the third lifetime component, yielding viscosities of the order expected for membranes [51]: ca. 536 cP (2.7 ns) for the high glucose condition, and ca. 869 cP (3.5 ns) for the low glucose condition. As all other variables between the two conditions (day of seeding, growth period, order and duration of imaging) have been controlled for, this provides convincing evidence that the FMR is indeed probing the mitochondrial membrane, and further that environmental cell conditions prior to extraction can affect membrane fluidity. These experiments show that even slight starvation can induce membrane rigidification which is consistent with lipid peroxidation due to increased ROS levels.

### Effect of FMR-2 on model lipid bilayer

**Fig 7:**
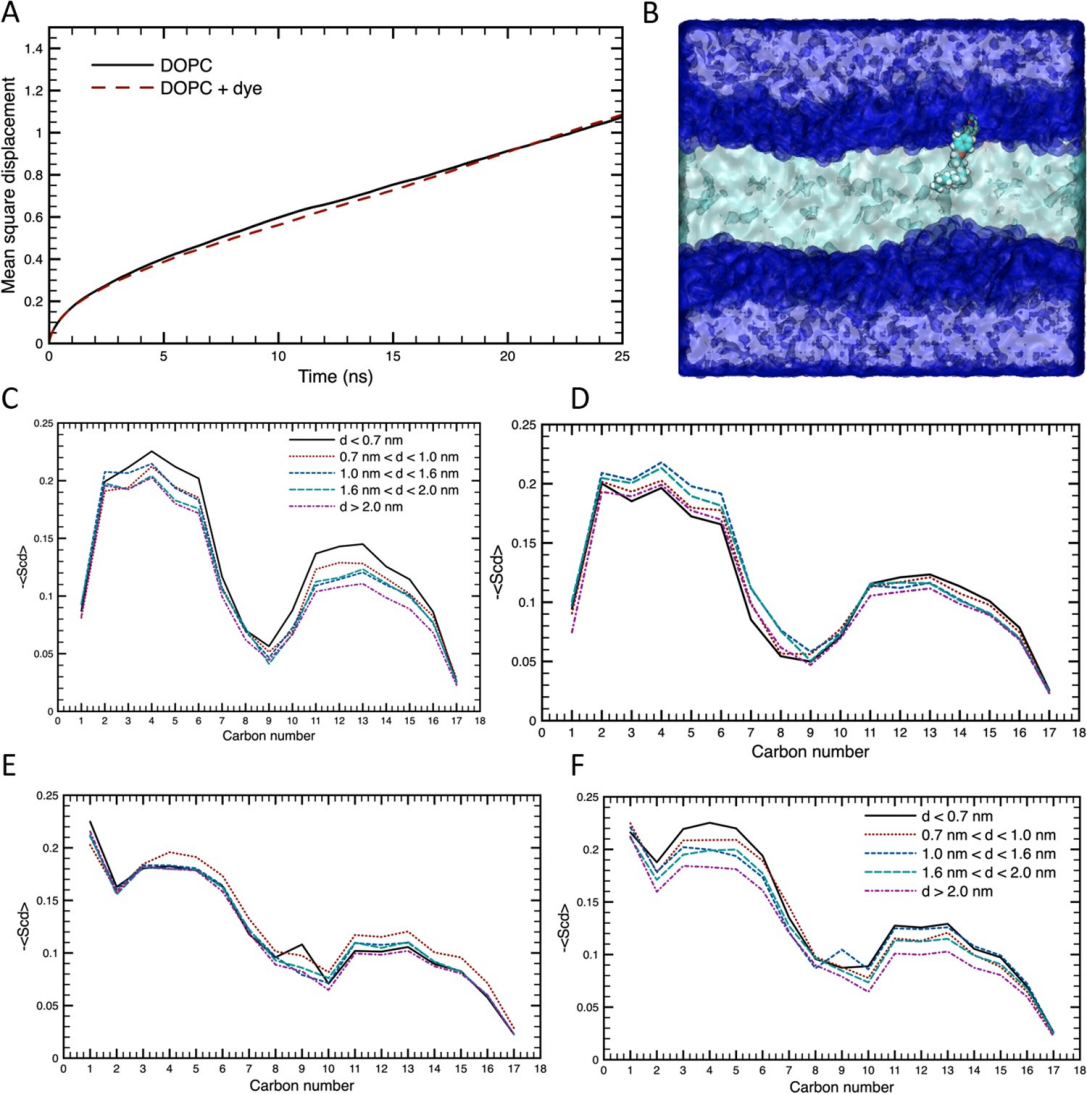
A) Mean square displacement of phosphorous atoms in the phosphatidylcholine headgroup of the lipid molecules in a DOPC bilayer with and without FMR-2. B) Snapshot of MD simulation showing position of **FMR-2** within the model lipid bilayer. C) Lipid order parameters for *sn*-1 in the same leaflet as **FMR-2**. D) Lipid order parameters for *sn*-1 in the opposite leaflet as **FMR-2**. E) Lipid order parameters for *sn*-2 in the same leaflet as **FMR-2**. F) Lipid order parameters for *sn*-2 in the opposite leaflet as **FMR-2**.

As **FMR-2** is not a flat molecule, we wanted to investigate the effect of the FMR on a membrane; specifically whether it could potentially bias our results by affecting the very property we are trying to probe. To that end, we used all-atom MD simulations to investigate the effect that the presence of **FMR-2** has on the dynamic, structural and interfacial properties of a DOPC bilayer. In order to assess the effect on the dynamic properties, we measured the mean square displacement (msd) of the phosphorous atoms in the phosphatidylcholine headgroup of the lipid molecules in a DOPC bilayer and a DOPC bilayer in which a **FMR-2** has adsorbed. Fig 7A shows the msd for the two different bilayers, and there is no significant difference in the two curves. Therefore, it appears that there is no effect on the diffusion of the lipids within the bilayer when the dye is present at this ratio of 1 dye molecule to 288 lipid molecules, as compared to when it is not present.

In order to assess any effect the dye molecule has on the structure of the lipid bilayer, we have measured the carbon-hydrogen lipid order parameter for lipid molecules whose phosphorous atom is within a given distance of the boron atom in the dye molecule. Fig 7C–7E shows the lipid order parameters for *sn*-1 and *sn*-2 chains in the same and opposite leaflets as the dye molecule. In doing so, we have shown these lipid order parameters for those lipids that are first neigbours of the dye molecule and then at increasing distances in the *x*-*y* plane from the dye molecule. In the same leaflet, we find that the *sn*-1 and *sn*-2 chains of the first neighbour lipid molecules (*d* < 0.7 nm) are slightly more ordered than those chains at the larger distances, but there is no effect on chains at any larger distances. While in the opposite leaflet, there is no significant difference in the order parameter as a function of distance from the dye.

Finally, we have determined the average number of first neighbour water molecules around the onium, phosphate and ester groups of the phosphatidylcholine head groups of lipids at different distances within the *x*-*y* plane from the dye molecule (as shown in Table 1). From these values, we find that the DOPC lipid molecules that are first neighbours of the dye molecule (*d* < 0.7 nm) in the same leaflet have their headgroups dehydrated by approximately 1 water molecule as compared to the headgroups at further distances. Meanwhile, there is no effect of the hydration of the headgroups of the lipid molecules in the opposite leaflet. Therefore, once again, at this ratio of dye to lipid molecules, it appears that the dye will have very minor effects on the interfacial properties of the lipid membranes.

**Table 1:** Average number of first neighbour water molecules.

	Onium group		Phosphate group		Ester groups	
Distance	Same	Opposite	Same	Opposite	Same	Opposite
d <0.7 nm	16.8 (4.0)	17.8 (3.8)	6.1 (1.4)	6.4 (1.4)	1.9 (1.0)	2.1 (1.0)
0.7 <d <1.0 nm	17.5 (3.8)	17.5 (3.8)	6.3 (1.4)	6.4 (1.4)	2.0 (1.0)	2.1 (1.0)
1.0 nm <d <1.6 nm	17.6 (3.8)	17.5 (3.8)	6.3 (1.4)	6.4 (1.4)	2.0 (1.0)	2.1 (1.0)
1.6 nm <d <2.0	17.4 (3.8)	17.4 (3.9)	6.3 (1.4)	6.3 (1.4)	2.1 (1.0)	2.1 (1.0)
d <2.0 nm	17.4 (3.9)	17.5 (3.9)	6.3 (1.4)	6.3 (1.4)	2.1 (1.0)	2.1 (1.0)

Average number of first neighbour water molecules around the onium, phosphate and ester groups of the phosphatidylcholine headgroups of the DOPC lipid molecules in the same and opposite leaflets as the **FMR-2** molecule at different distances in the *x*-*y* plane from the dye molecule. The standard deviation of the values are shown in parentheses.

## Discussion

In this paper, we explored the effect of increased Ca^2+^ exposure on matrix viscosity and slight nutrient deprivation on membrane fluidity. In both cases, viscosity/fluidity exhibited variation, showing that these parameters are dynamic and not “fixed”, even in healthy cells—organelles may be constantly responding to environmental and physiological fluctuations. We believe this warrants further investigation because we cannot describe pathological states without first understanding the range of viscosity variation in healthy controls. It is also important to understand that, in cases where the viscosity/rigidity increases, diffusion-limited reactions will be slowed down. Conversely, higher fluidity would likely cause a rate increase. This could have potential knock-on effects for the whole organelle, cell and potentially organism. Changes in viscosity could therefore play a significant role in the mechanism behind certain illnesses; this again underlines the need to understand the physiological viscosity state. Further, it could be relevant for drug delivery systems that inherently rely on diffusion. From a methodological perspective, we have shown that a combination of chemical targeting and organelle extraction enables easy imaging of organelle viscosity and membrane fluidity. To the best of our knowledge, this is the first fully combined framework for imaging both matrix viscosity and membrane fluidity using FMR-FLIM.

To thoroughly check that **FMR-1** is only sensitive to viscosity, we have carried out a series of control experiments, studying the effects of temperature, polarity and ionic strength in turn. We find that, while some of these measures may alter the viscosity, they are not otherwise related—the FMR reports on viscosity and nothing else. This is completely in line with the findings of others on the topic of BODIPY FMRs [43],[44]. **FMR-2** has already been used to study membrane fluidity and membrane order in binary and ternary lipid mixtures, as well as membrane cholesterol content, in what has been termed ‘molecular rheometry’ [52],[53]. One such study found that, using **FMR-2**, the transition temperature of DPPC could be confirmed, and that the reported viscosity values were in the expected range [52]. However, to test whether any effect of **FMR-2** on the membrane it was probing could possibly bias our results, we have carried out MD simulations to explore a model lipid bilayer with and without **FMR-2**. Our findings show that the presence of the dye has no significant impact upon lipid diffusion, and that only very minor changes in lipid order and headgroup hydration are induced, and only at very short distances from the dye. We find this convincing evidence that **FMR-2** can be used to study membrane fluidity at low concentrations without significant perturbation of the system studied.

As mentioned, some other methods allow probing of viscosity and the related concept of diffusion. One is FCS, which measures the intensity fluctuations of a small concentration of dye molecules diffusing through a volume to gain diffusion information [54]. However it requires significant knowledge of the studied system and extremely low dye concentrations (pico- to nanomolar range) [54]. Another method, FRAP, includes bleaching a region of interest and measuring the repopulation time [55]. In biological samples, the effect of organelle shape and geometric boundaries can limit “back-diffusion” and hence recovery [56],[57], of particular importance in mitochondria because of their cristae. FRAP cannot be used to create “diffusion maps” analogous to the viscosity maps created by FMR-FLIM. Finally, Generalized Polarization (GP) of suitable polarity-sensitive membrane dyes, which exploits a spectral shift in emission between gel and liquid lipid phases, can be used to study membrane lipid order [58][59]. Rather than measuring viscosity, it is based on sensing the penetration of highly polar water molecules into the membrane. GP images are obtained by imaging in two spectral regions, and is widely used to specifically study membranes.

The main alternative to FMR-FLIM is anisotropy—specifically Time-Resolved Anisotropy Imaging Microscopy (TR-FAIM) [11]. TR-FAIM is usually conducted with a rigid dye molecule and the method can indicate hindered rotation. However, anisotropy decays can be very noisy, and they require high photon counts to fit properly—upwards of 1000 photons in each pixel. In contrast, counts in the low hundreds can be used for FLIM [60],[61]. As a result, much longer acquisition times are needed for scanning TCSPC TR-FAIM; this is problematic for biological samples, dynamic events and/or photobleaching. It also means that FMR-FLIM has bigger potential for viscosity movies than TR-FAIM. Finally, in contrast to FLIM, TR-FAIM lacks commercial analysis software (manual analysis is computationally cumbersome and non-trivial). Therefore, FMR-FLIM represents an easier, quicker alternative to TR-FAIM, especially for non-specialists.

Clinical studies may in particular benefit from FMR-FLIM. We chose to focus on various mitochondrial regions in part because the diffusion and fluidity of these have been the subject of intense clinical research. Altered protein diffusion in the mitochondrial matrix has been linked to Complex I deficiency [4], and changes in mitochondrial membrane fluidity have been found in the mitochondria of Alzheimer’s disease patients [2], as well as Huntington’s, Alzheimer’s and Amyotrophic lateral sclerosis mouse models [3]. FMR-FLIM could uncover new insights in this area, underlining the wide range of potential applications for this technique.

## Conclusion

Cellular viscosity imaging has so far lacked a framework for imaging both matrix/lumenal viscosity and membrane fluidity of organelles. In mitochondria, we have shown that the combination of chemical FMR targeting, organelle extraction and FMR-FLIM allow us to image the viscosity of the major regions within the organelles, namely the matrix and the membranes. We have shown that these properties are dynamic and responsive to small environmental and physiological changes, and further, we have tested the suitability of FMR-FLIM in membranes through MD simulation. We believe that single-organelle FMR-FLIM has the potential to find widespread use in biophysical, clinical and drug delivery studies.

## Supporting information

S1 Fig All spectra refer to FMR-1. For similar details on **FMR-2**, see previous publication [26]. A) Fluorescence emission spectra with increasing viscosity (10% to 80% glycerol, 1.3 cP to 380 cP). B) Peak emission intensity plotted against viscosity in a log-log plot. The gradients of the low and high viscosity regions are 0.25 and 0.64, respectively. C) Quantum yield plotted against viscosity in a log-log plot. The gradients of the low and high viscosity regions are 0.25 and 0.64, respectively, in excellent agreement with B. Note that the gradients of the fitted regions for both log-log plots are in excellent agreement with the gradients achieved for the log-log lifetime/viscosity plot (see main text), confirming that **FMR-1** is perfectly in line with theory D) Steady-state excitation anisotropy, measured at the main emission feature at 525 nm, against wavelength with viscosity increasing from 55% to 80% glycerol. It shows the characteristic dip-and-rise anisotropy of FMRs [13]. The negative feature from roughly 380 to 490 nm is due to S_0_-S_2_ excitation, where the absorption transition is roughly orthogonal to the emission dipole [13]. Past 460 nm the anisotropy increases with viscosity.
(TIF)

S2 Fig A-D) Representative intensity and FLIM images of HeLa cells treated with an increasing concentration of histamine (10*μ*M, 20*μ*M, 30*μ*M, 40*μ*M respectively). To the left of the images are representative decays with fit and scaled IRF. E) Boxplot showing control (n = 140) and the 10*μ*M (n = 57), 20*μ*M (n = 35) histamine conditions respectively. F) Boxplot showing control (n = 140) and the 40*μ*M (n = 120) condition.
(TIF)

S3 Fig ^1^H NMR spectrum of FMR-1 in deuterated chloroform. (TIF)

S4 Fig ^13^C NMR spectrum of FMR-1 in deuterated chloroform. (TIF)

S5 Fig ^19^F NMR spectrum of FMR-1 in deuterated chloroform. (TIF)

S6 Fig Mass spectroscopy of FMR-1. (TIF)

S7 Fig Closer view of mass spectroscopy of FMR-1. S6 Fig and S7 Fig clearly show the relevant m/z peak at 601.2384 [M-Br], matching the calculated mass for C_37_H_33_BF_2_N_2_OP, 601.2392.
(TIF)

S8 Fig A) Intensity and FLIM images of HCE cells at 37°C and 5% CO_2_. Staining protocol was the same as the one described for HeLa cells. B) Representative decay from the FLIM image with scaled IRF, fit and residuals. C) Normalised frequency histogram for all of the image in A, clearly showing a Gaussian distribution. To show that FMR-1 performs equally under more physiologically relevant conditions, we tested it in HCE cells, a human, non-cancerous epithelial cell line, at physiologically relevant temperature and CO_2_ levels. S8 Fig shows that here too, we can collect FLIM images and decays for easy analysis.
(TIF)

S1 File Brief description of the underlying theory of FMRs and their calibration. (PDF)
