# Supplementary figures and images for: Targeted fluorescence lifetime probes reveal responsive organelle viscosity and membrane fluidity
Source: PLoS One. 2019 Feb 14;14(2):e0211165. doi: 10.1371/journal.pone.0211165 (PMC6375549; doi:10.1371/journal.pone.0211165)

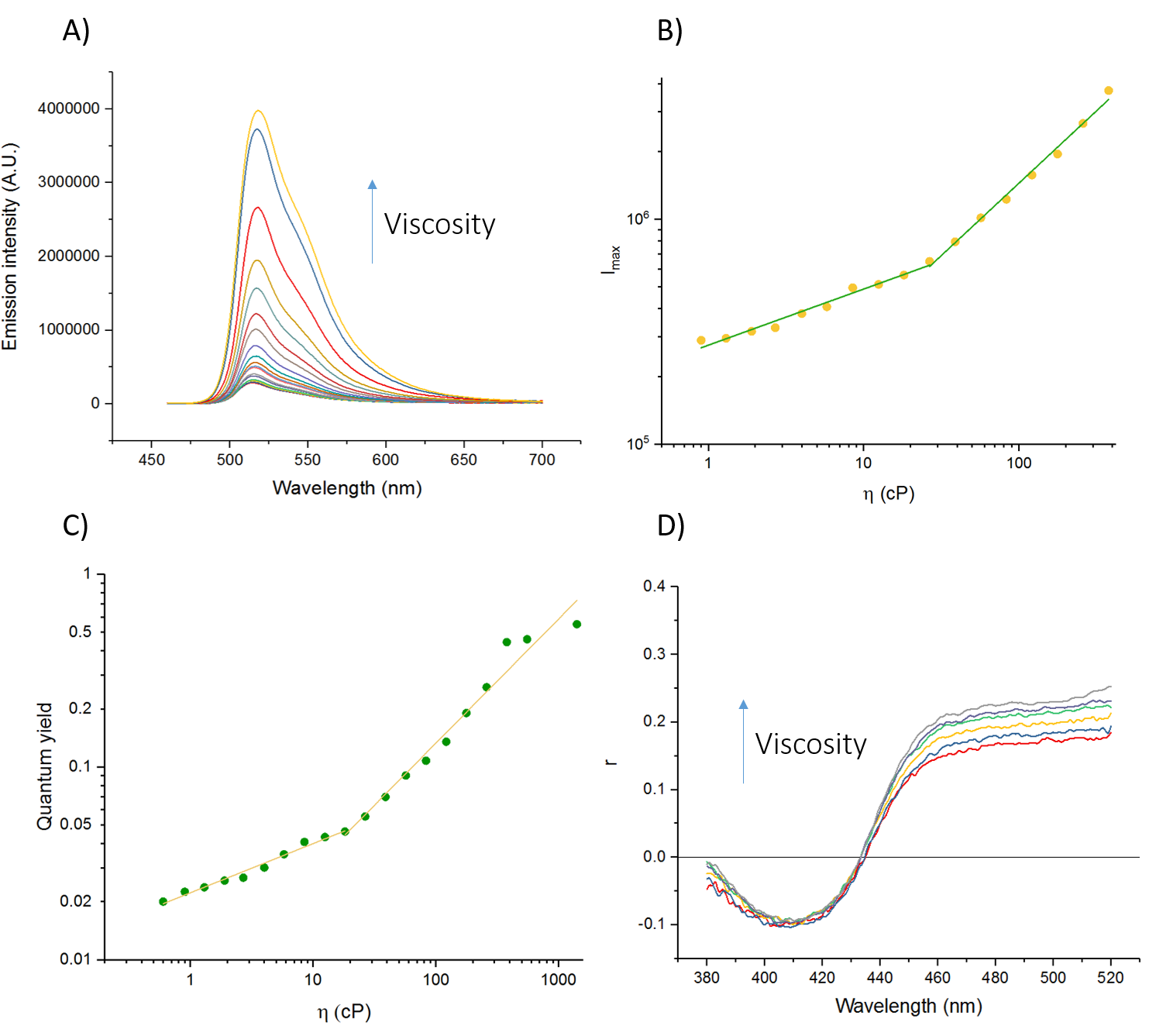

Supplement: S1 Fig — For similar details on FMR-2, see previous publication [26]. A) Fluorescence emission spectra with increasing viscosity (10% to 80% glycerol, 1.3 cP to 380 cP). B) Peak emission intensity plotted against viscosity in a log-log plot. The gradients of the low and high viscosity regions are 0.25 and 0.64, respectively. C) Quantum yield plotted against viscosity in a log-log plot. The gradients of the low and high viscosity regions are 0.25 and 0.64, respectively, in excellent agreement with B. Note that the gradients of the fitted regions for both log-log plots are in excellent agreement with the gradients achieved for the log-log lifetime/viscosity plot (see main text), confirming that FMR-1 is perfectly in line with theory D) Steady-state excitation anisotropy, measured at the main emission feature at 525 nm, against wavelength with viscosity increasing from 55% to 80% glycerol. It shows the characteristic dip-and-rise anisotropy of FMRs [13]. The negative feature from roughly 380 to 490 nm is due to S0-S2 excitation, where the absorption transition is roughly orthogonal to the emission dipole [13]. Past 460 nm the anisotropy increases with viscosity. (TIF) [file pone.0211165.s001.tif]

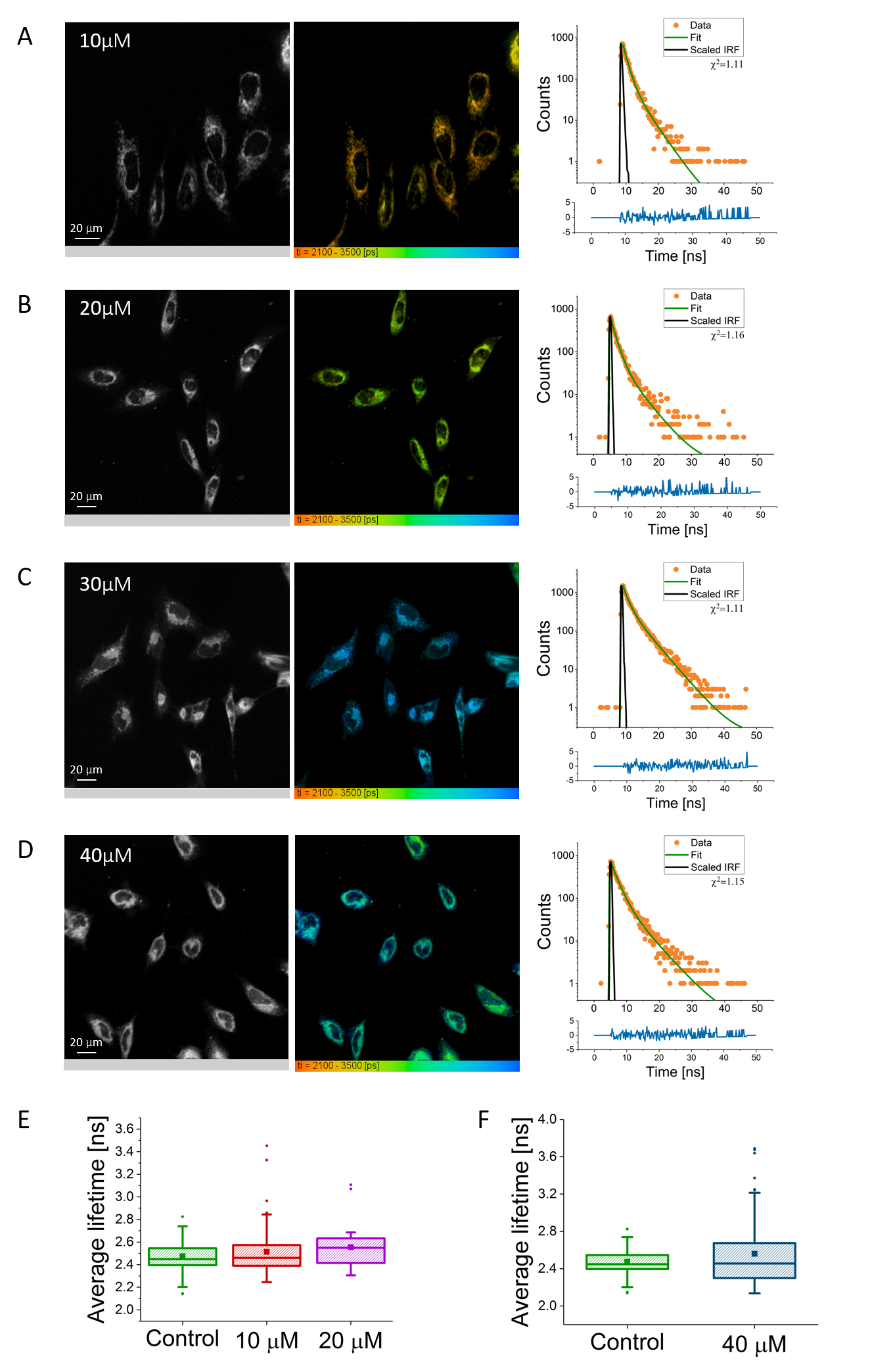

Supplement: S2 Fig — A-D) Representative intensity and FLIM images of HeLa cells treated with an increasing concentration of histamine (10μM, 20μM, 30μM, 40μM respectively). To the left of the images are representative decays with fit and scaled IRF. E) Boxplot showing control (n = 140) and the 10μM (n = 57), 20μM (n = 35) histamine conditions respectively. F) Boxplot showing control (n = 140) and the 40μM (n = 120) condition. (TIF) [file pone.0211165.s002.tif]

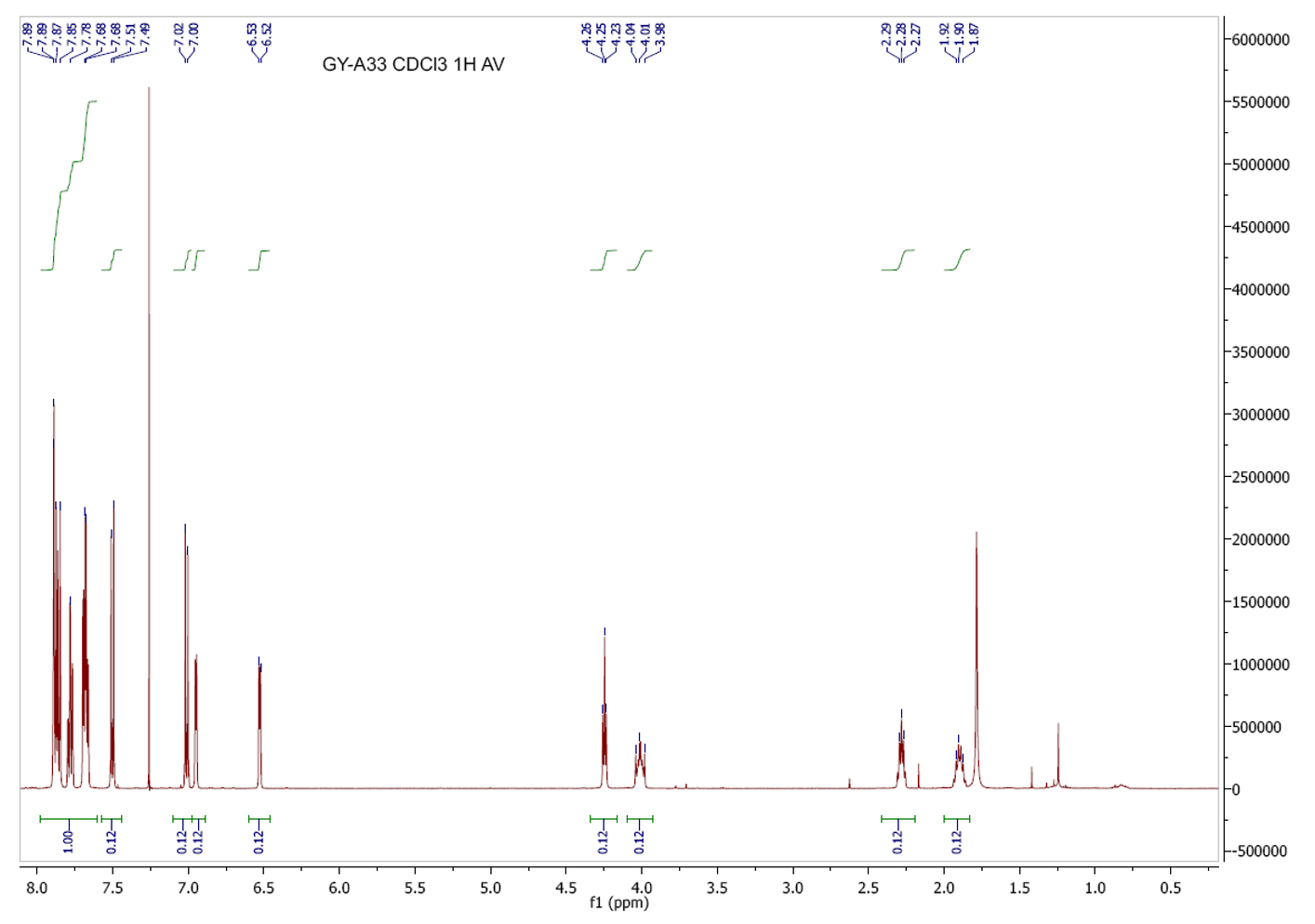

Supplement: S3 Fig — (TIF) [file pone.0211165.s003.tif]

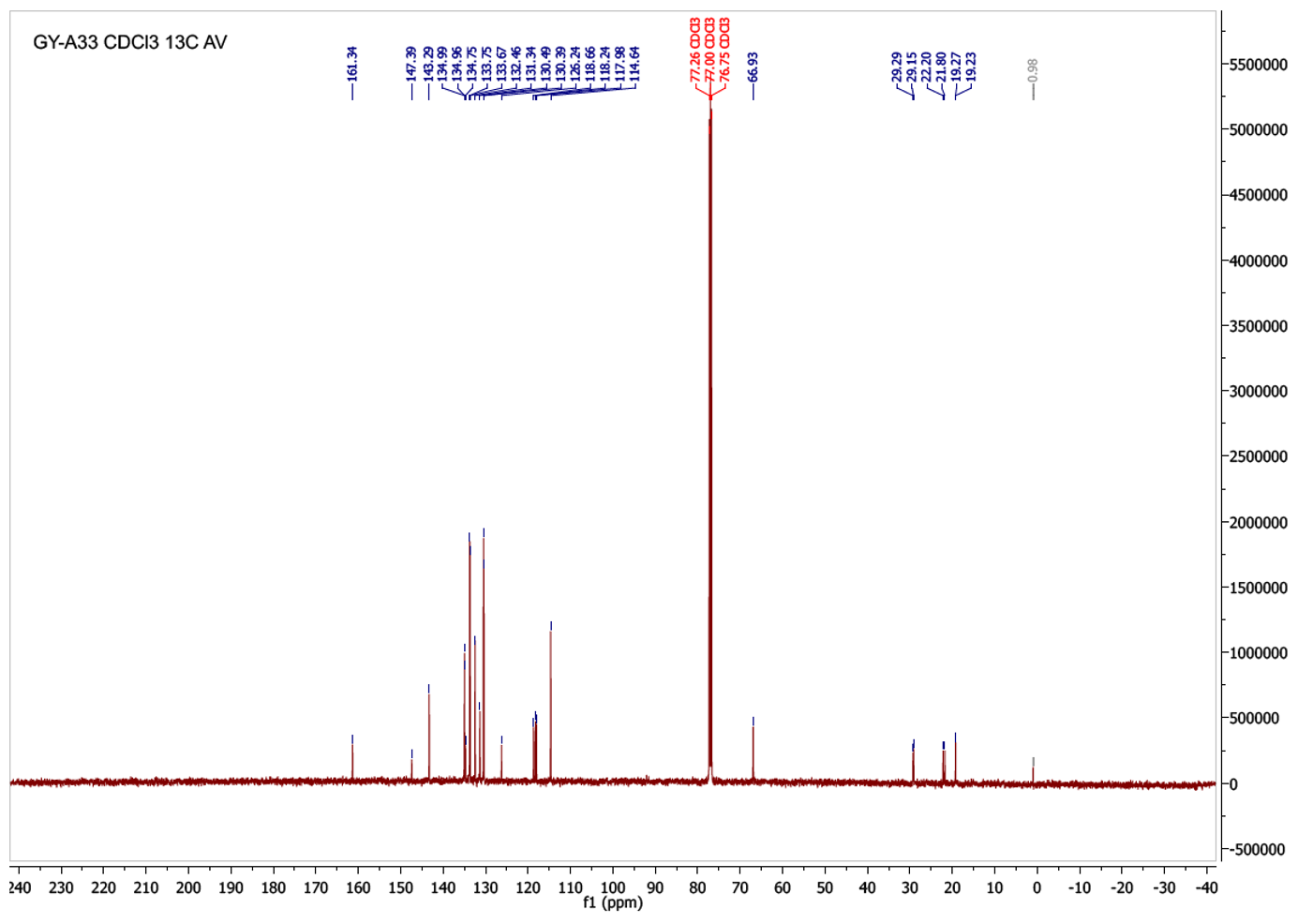

Supplement: S4 Fig — (TIF) [file pone.0211165.s004.tif]

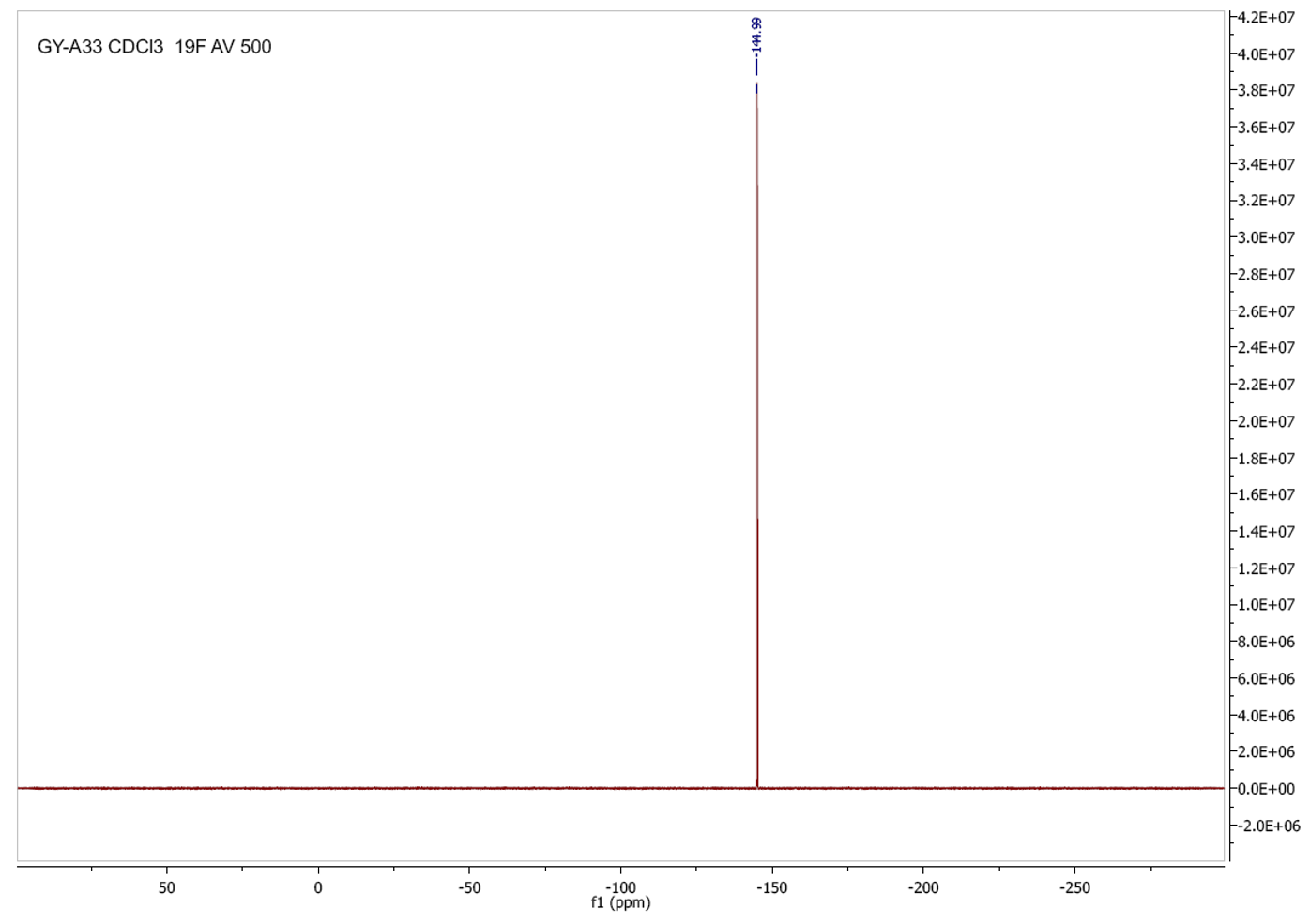

Supplement: S5 Fig — (TIF) [file pone.0211165.s005.tif]

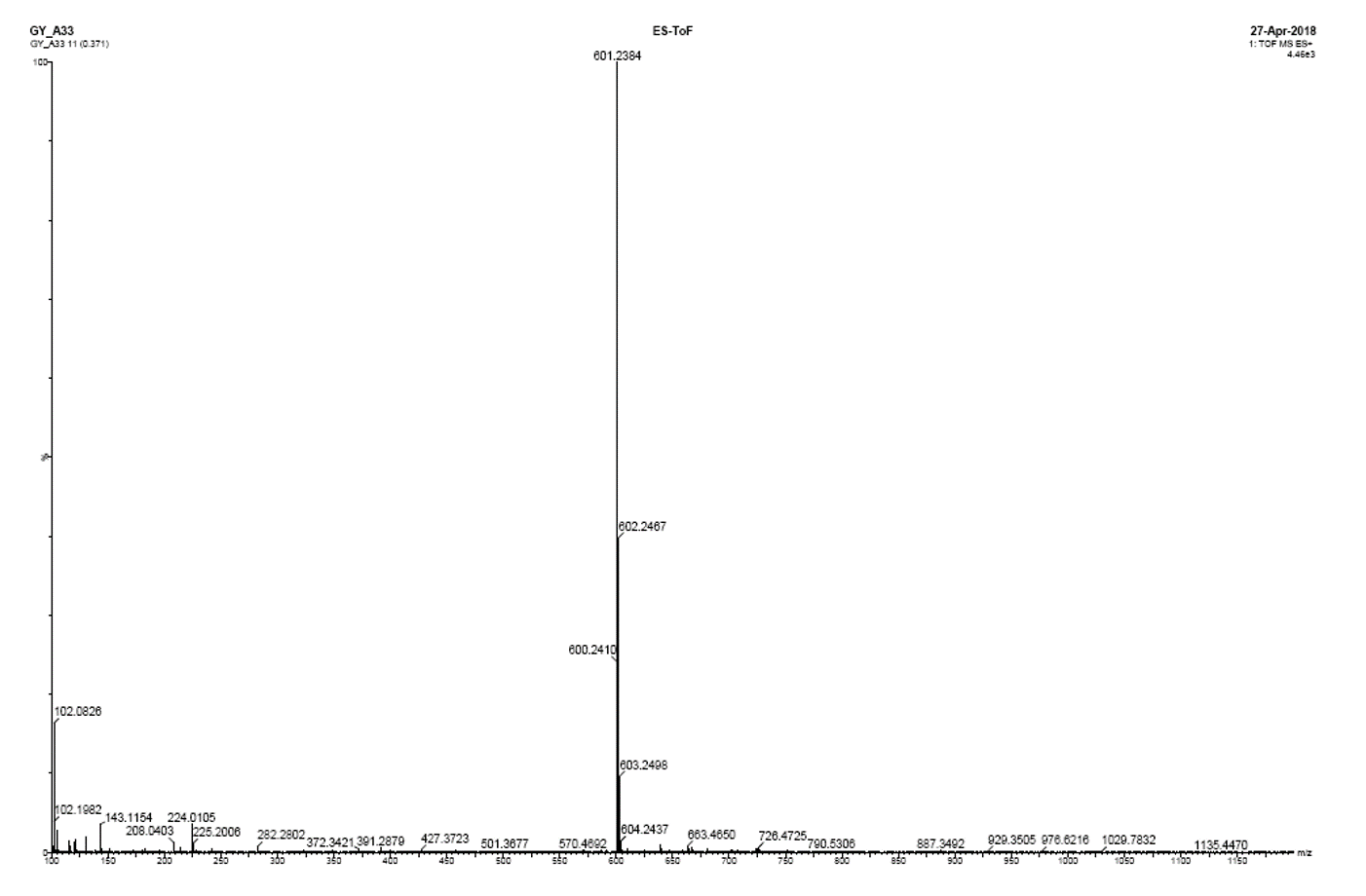

Supplement: S6 Fig — (TIF) [file pone.0211165.s006.tif]

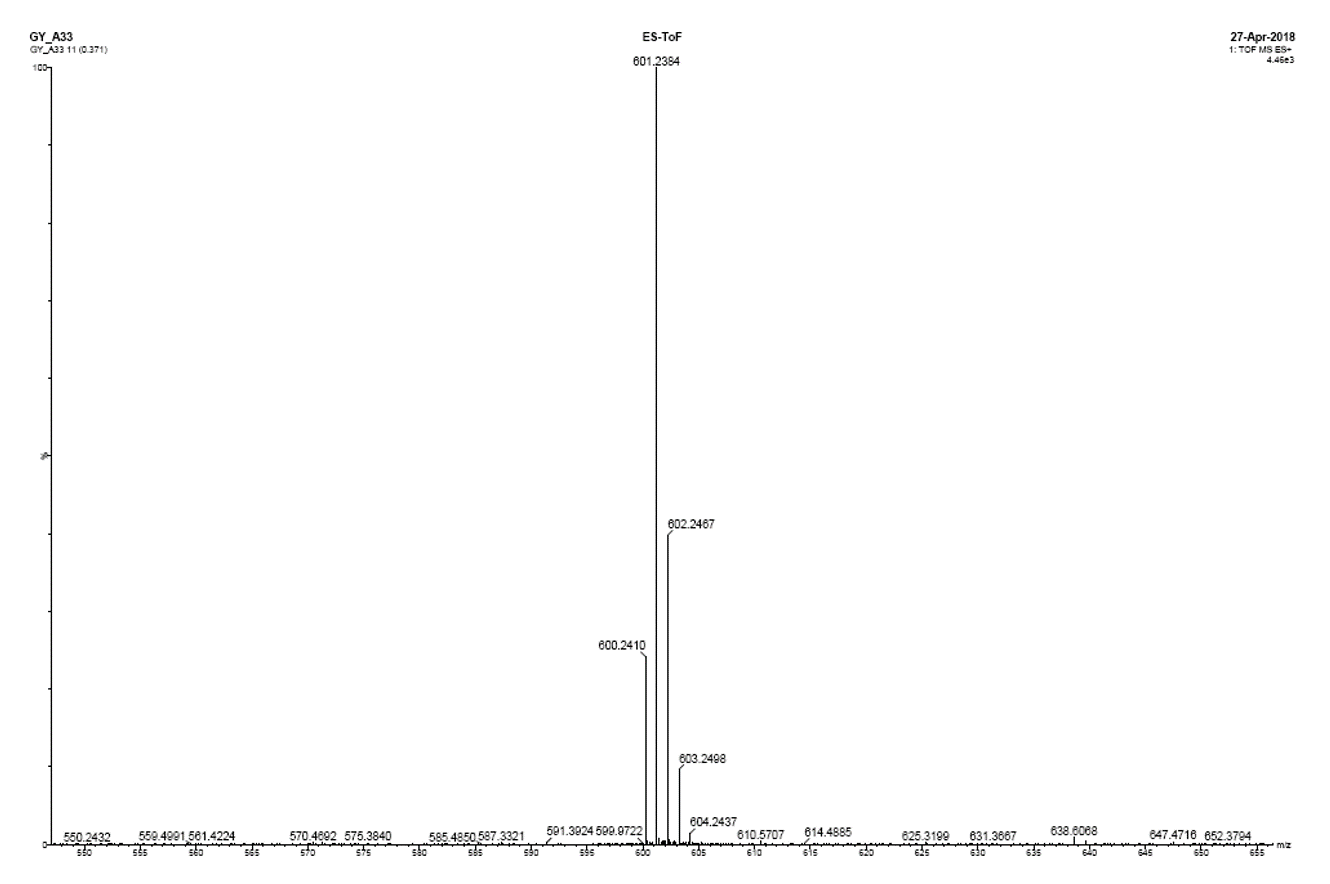

Supplement: S7 Fig — S6 Fig and S7 Fig clearly show the relevant m/z peak at 601.2384 [M-Br], matching the calculated mass for C37H33BF2N2OP, 601.2392. (TIF) [file pone.0211165.s007.tif]

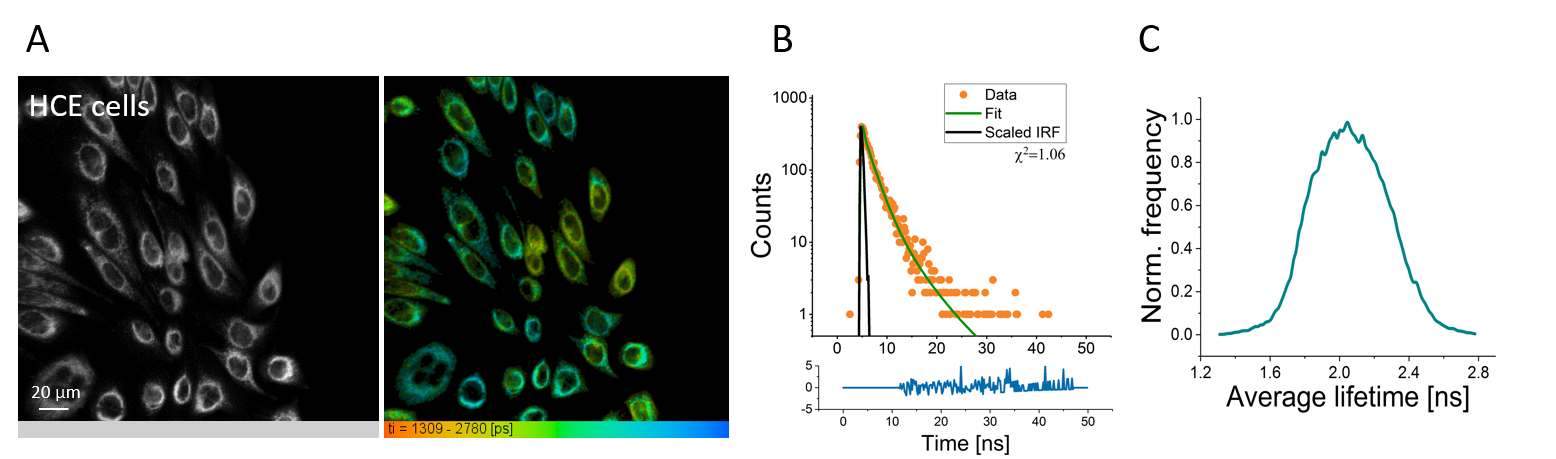

Supplement: S8 Fig — A) Intensity and FLIM images of HCE cells at 37°C and 5% CO2. Staining protocol was the same as the one described for HeLa cells. B) Representative decay from the FLIM image with scaled IRF, fit and residuals. C) Normalised frequency histogram for all of the image in A, clearly showing a Gaussian distribution. To show that FMR-1 performs equally under more physiologically relevant conditions, we tested it in HCE cells, a human, non-cancerous epithelial cell line, at physiologically relevant temperature and CO2 levels. S8 Fig shows that here too, we can collect FLIM images and decays for easy analysis. (TIF) [file pone.0211165.s008.tif]
