# Supplementary material for: Targeted fluorescence lifetime probes reveal responsive organelle viscosity and membrane fluidity
Source: PLoS One. 2019 Feb 14;14(2):e0211165. doi: 10.1371/journal.pone.0211165 (PMC6375549; doi:10.1371/journal.pone.0211165)
Supplement: S1 File — (PDF) [file pone.0211165.s009.pdf]

Supporting Information: Targeted fluorescence lifetime probes reveal responsive organelle viscosity and membrane fluidity

Ida Emilie Steinmark[1*], Arjuna L James[1], Pei-Hua Chung[1], Penny E Morton[2], Maddy Parsons[2], Cécile A Dreiss[3], Christian D Lorenz[1], Gokhan Yahioglu[4*,¤], Klaus Suhling[1*]

1 Department of Physics, King's College London, London, UK
2 Randall Centre for Cell and Molecular Biophysics, King's College London, London, UK
3 Institute of Pharmaceutical Science, King's College London, London, UK
4 Department of Chemistry, Imperial College London, London, UK

¤Current Address: Antikor Biopharma, Stevenage, UK
* ida.steinmark@kcl.ac.uk, klaus.suhling@kcl.ac.uk, g.yahioglu@antikor.co.uk

Theory: fluorescent molecular rotors and viscosity

In order to use FMRs to report on viscosity, a solid relationship between viscosity and FMR fluorescence is needed for proper calibration. In general, FMR papers describe the relationship between fluorescence quantum yield and viscosity through the Förster-Hoffman equation[1]:

$$\phi_f = z\eta^{\alpha} \tag{1}$$

This equation is based on the Debye-Stokes-Einstein theory of diffusion[2][3], and in the Förster-Hoffman paper, α is found to be $\frac{2}{3}$[4]. If the logarithm of both sides are taken, this provides a straight-line plot, which fitted Förster and Hoffman's data on Crystal Violet for three orders of magnitude, with some deviation at higher viscosities. Regardless, it provides an empirical relationship. Comparing with the equation for quantum yield, a lifetime version of the equation can be derived:

$$\phi_f = \frac{k_r}{k_r + k_{nr}} = \tau \times k_r \tag{2}$$

$$\tau = \frac{z}{k_r}\eta^{\alpha} \tag{3}$$

$$log\tau = log\frac{z}{k_r} + \alpha \times log\eta \tag{4}$$

Where the final equation yields a straight line – a similar equation can be constructed for fluorescence intensity. This roughly works for an observational calibration from which to convert lifetimes into viscosity values. It is worth noting that the Förster-Hoffman relationship only hold within a certain, intermediate range which will vary depending on the FMR. For BODIPY FMRs, this is roughly between 15 and 1000 cP for methanol/glycerol mixtures[2]. For calibration purposes, straight lines to different regions can be fitted for more accurate viscosity calculations.

References

1. Levitt JA, Chung PH, Kuimova MK, Yahioglu G, Wang Y, Qu J, et al. Fluorescence anisotropy of molecular rotors. ChemPhysChem. 2011;12(3):662–672.

2. Kuimova MK. Mapping viscosity in cells using molecular rotors. Physical Chemistry Chemical Physics. 2012;14(37):12671–12686.

3. Haidekker MA, Theodorakis EA. Environment-sensitive behavior of fluorescent molecular rotors. Journal of Biological Engineering. 2010;4(1):11.

4. Förster T, Hoffmann G. Die Viskositätsabhängigkeit der Fluoreszenzquantenausbeuten einiger Farbstoffsysteme. Zeitschrift für Physikalische Chemie. 1971;75(1_2):63–76.
